# Supplementary material for: The actin nucleation promoting factor WASH facilitates clathrin-independent endocytosis of human papillomaviruses
Source: EMBO Rep. 2025 Oct 10;26(22):5533–66. doi: 10.1038/s44319-025-00594-3 (PMC12635285; doi:10.1038/s44319-025-00594-3)
Supplement: Supplementary file 5 — Expanded View Figures [file 44319_2025_594_MOESM5_ESM.pdf]

Expanded View Figures

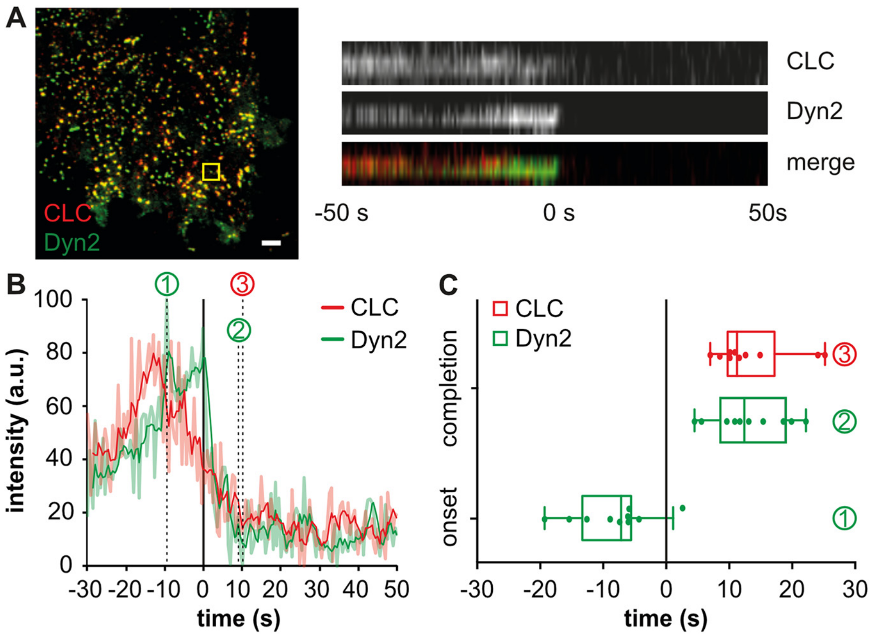

Figure EV1. Kinetics of dynamin recruitment during CME.

(**A**) HeLa ATCC cells were co-transfected with mRFP-clathrin light chain (CLC) and EGFP-dynamin 2 (Dyn2). Cells were imaged by live-cell TIRF-M. Movies were acquired with 0.5 Hz frame rate for 5 min. CME events denoted by CLC signal loss were identified manually after background subtraction and filtering. The yellow box indicates the CME event shown as kymograph. Scale bar is 2 µm. (**B**) Plotted are the intensity profiles of CLC and Dyn (light red/green) as well as moving averages (intense red/green). Note that due to its additional role in vesicle maturation, Dyn2 was already present early during vesicle formation (Loerke et al, 2009; Taylor, Lampe and Merrifield, 2012). A second wave of recruitment was observed for scission and quantified in (**C**). (**C**) The onset of Dyn2 recruitment for scission (1) relative to the half-time of CLC loss from the cell surface ($t = 0$) as well as the timepoint of the completion of CLC (3) and Dyn2 (2) signal loss were manually determined from intensity profiles. Data information: For (**C**), depicted are values from 10 profiles (from $n = 3$ biological replicates). Dots in the bar graphs indicate the individual endocytosis events. Box plots indicate the interquartile range (25th to 75th percentiles, box) and median, while the whiskers extend to the minimum and maximum values of all data points.

 © The Author(s)

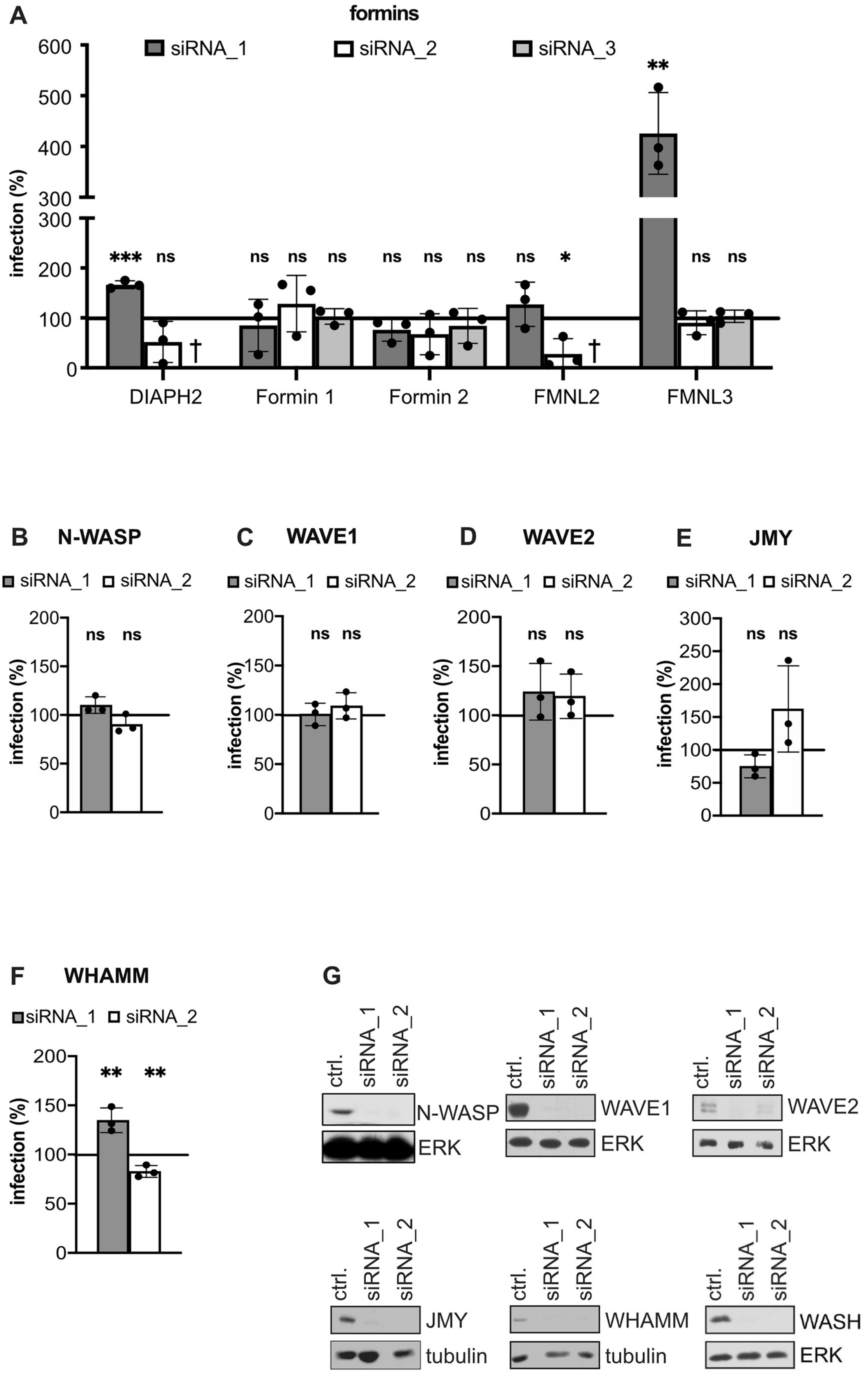
A
formins
siRNA_1
siRNA_2
siRNA_3
infection (%)
600
500
400
300
300
200
100
0
**
***
ns
*
†
DIAPH2
Formin 1
Formin 2
FMNL2
FMNL3
B
N-WASP
C
WAVE1
D
WAVE2
E
JMY
F
WHAMM
G
ctrl.
siRNA_1
siRNA_2
N-WASP
ERK
WAVE1
WAVE2
JMY
tubulin
WHAMM
WASH

Figure EV2. Unbranched actin polymerization regulated by formins, nucleation-promoting factors N-WASP, WAVE, WHAMM and JMY are dispensable for HPV16 infection.

(**A**) HeLa Kyoto cells were depleted of individual formins and infected with HPV16-GFP. Infection was scored 48 h p.i. by automated microscopy and normalized to ctrl., siRNAs that reduced cell numbers by more than 80% were considered cytotoxic (†) and excluded from the analysis. Dots in the bar graphs indicate the replicates. (**B–F**) HeLa Kyoto cells were infected with HPV16-GFP after siRNA mediated depletion of N-WASP (**B**), WAVE1 (**C**), WAVE2 (**D**), JMY (**E**), WHAMM (**F**). Infection was scored 48 h p.i. by automated microscopy, normalized to ctrl. ($n = 3$ biological replicates). Dots in the bar graphs indicate the replicates. (**G**) Protein expression levels were determined by Western blotting against the siRNA target proteins. Data information: For (**A–F**) data are represented as mean ± SD of $n = 3$ biological replicates. For all quantifications, statistical significance was assessed by Student's *t* test (*$P \leq 0.05$, **$P \leq 0.01$, ***$P \leq 0.001$, ****$P \leq 0.0001$, ns = not significant). In (**A**), significance values in comparison to siRNA ctrl.: DIAPH2: siRNA_1: $P = 0.0001$, siRNA_2: $P = 0.1121$; Formin 1: siRNA_1: $P = 0.6476$, siRNA_2: $P = 0.4332$, siRNA_3: $P = 0.7612$; Formin 2: siRNA_1: $P = 0.1417$, siRNA_2: $P = 0.2425$, siRNA_3: $P = 0.4725$; FMNL2: siRNA_1: $P = 0.3478$, siRNA_2: $P = 0.0149$; FMNL3: siRNA_1: $P = 0.0022$, siRNA_2: $P = 0.5163$, siRNA_3: $P = 0.6605$. In (**B**), significance values in comparison to siRNA ctrl.: N-WASP: siRNA_1: $P = 0.0672$, siRNA_2: $P = 0.247$. In (**C**), significance values in comparison to siRNA ctrl.: WAVE1: siRNA_1: $P = 0.9101$, siRNA_2: $P = 0.3006$. In (**D**), significance values in comparison to siRNA ctrl.: WAVE2: siRNA_1: $P = 0.2236$, siRNA_2: $P = 0.2144$. In (**E**), significance values in comparison to siRNA ctrl.: WHAMM: siRNA_1: $P = 0.0087$, siRNA_2: $P = 0.0078$. In (**F**), significance values in comparison to siRNA ctrl.: JMY: siRNA_1: $P = 0.069$, siRNA_2: $P = 0.1747$.

 © The Author(s)

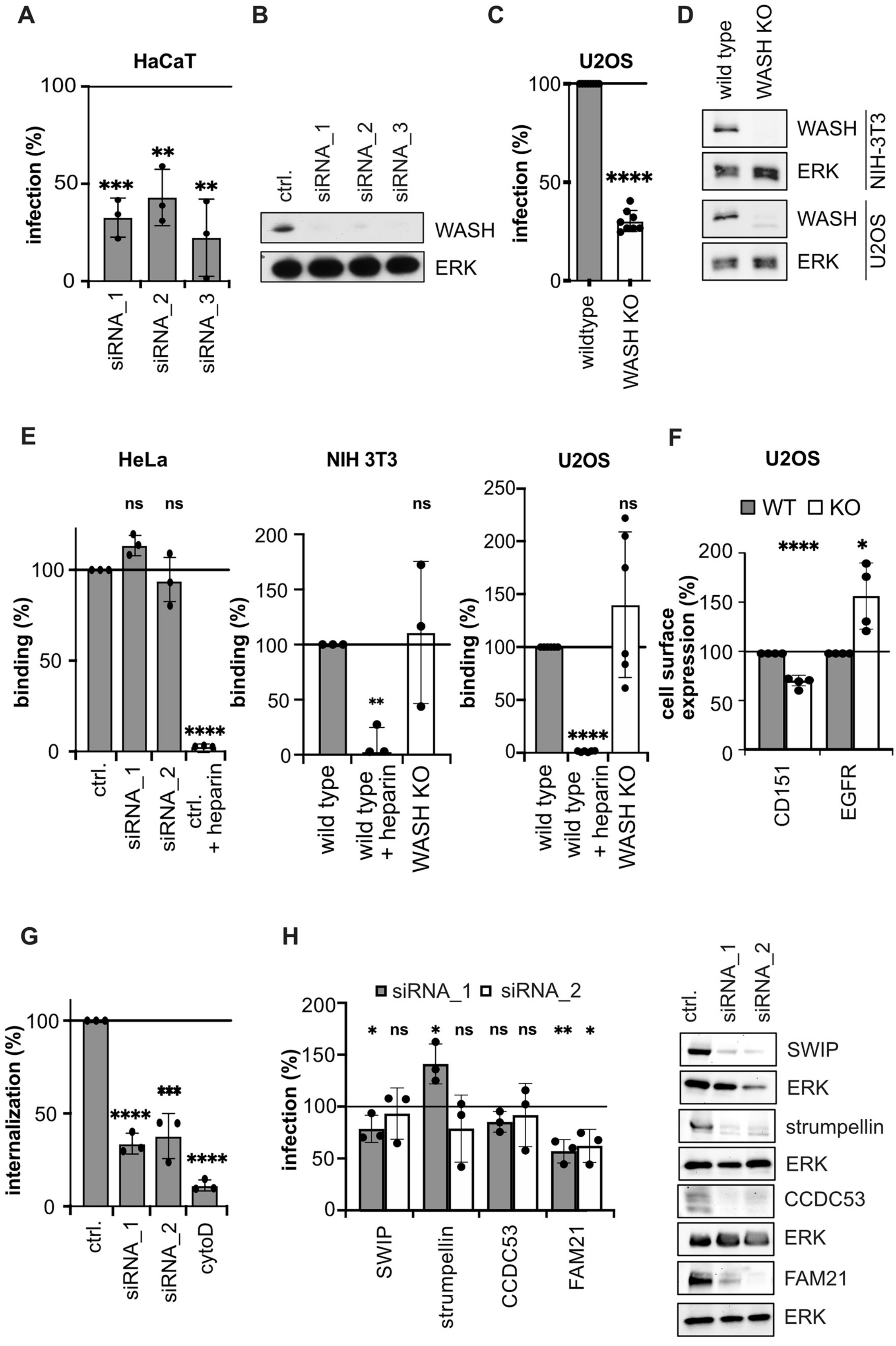
A
HaCaT
infection (%)
100
50
0
***
**
**
siRNA_1
siRNA_2
siRNA_3
B
ctrl.
siRNA_1
siRNA_2
siRNA_3
WASH
ERK
C
U2OS
infection (%)
100
50
0
****
wildtype
WASH KO
D
wild type
WASH KO
WASH
ERK
NIH-3T3
WASH
ERK
U2OS
E
HeLa
ns
ns
binding (%)
100
50
0
****
ctrl.
siRNA_1
siRNA_2
ctrl. + heparin
NIH 3T3
ns
binding (%)
200
150
100
50
0
**
wild type
wild type + heparin
WASH KO
U2OS
ns
binding (%)
250
200
150
100
50
0
****
wild type
wild type + heparin
WASH KO
F
U2OS
WT
KO
****
*
cell surface expression (%)
200
150
100
50
0
CD151
EGFR
G
internalization (%)
100
50
0
****
***
****
ctrl.
siRNA_1
siRNA_2
cytoD
H
siRNA_1
siRNA_2
* ns * ns ns ns ** *
infection (%)
200
150
100
50
0
SWIP
strumpellin
CCDC53
FAM21
ctrl.
siRNA_1
siRNA_2
SWIP
ERK
strumpellin
ERK
CCDC53
ERK
FAM21
ERK

Figure EV3. Further characterization and analysis of WASH perturbation.

(**A**) HaCaT cells were depleted of WASH by RNAi and subsequently infected with HPV16-GFP for 48 h. Infection levels were analyzed by automated microscopy and normalized to siRNA ctrl. (**B**) WASH depletion in HaCaT of (**A**) was confirmed by immunoblotting against WASH. (**C**) U2OS wild-type and WASH KO cells were infected with HPV16-GFP. Infection was analyzed 48 h p.i. by flow cytometry and normalized to wild-type cells. (**D**) WASH CRISPR/Cas9 KO in NIH-3T3 and U2OS KO cells was confirmed by immunoblotting against WASH in comparison to WT cells. (**E**) HPV16-AF488 was bound to Hela Kyoto cells transfected with ctrl. or WASH targeting siRNAs, NIH-3T3 or U2OS wild-type and WASH KO cells as indicated. Virus pre-incubated with 1 mg/ml heparin was used as a non-binding control. At 2 h p.i., virus binding was measured by flow cytometry, or in case for U2OS cells by automated microscopy. Values are shown as relatives to wild-type cells. (**F**) U2OS wild-type and WASH KO cells were detached by EDTA treatment. Extracellular epitopes of CD151 and EGFR were immunostained and cell surface levels were determined as the geometric mean intensity by flow cytometry. Values were normalized to wild-type cells. (**G**) HPV16-AF594 was bound to Hela Kyoto cells transfected with ctrl. or WASH targeting siRNAs and allowed to enter for 6 h. As control inhibiting uptake, cytochalasin D was used (10 µg/ml). The fluorescence of extracellular virus was quenched using trypan blue, and the fluorescence intensity of intracellular virus was analyzed by flow cytometry. Values are shown as relatives to ctrl. siRNA transfected cells. (**H**) HeLa Kyoto cells were depleted of SHRC proteins and infected with HPV16-GFP. Infection levels were analyzed by automated microscopy and normalized to ctrl. Protein levels were determined by Western blotting. Data information: In (**A**, **C**, **E**–**H**) data are represented as mean ± SD. For all quantifications, statistical significance was assessed by Student's *t* test excepting (**E**, HeLa), where a Welsh's *t* test has been used (*$P \le 0.05$, **$P \le 0.01$, ***$P \le 0.001$, ****$P \le 0.0001$, ns = not significant). In (**A**), data of $n = 3$ biological replicates. Significance values in comparison to siRNA ctrl.: siRNA_1: $P = 0.0003$, siRNA_2: $P = 0.0024$, siRNA_3: $P = 0.0025$. In (**C**), data of $n = 8$ biological replicates. Significance value in comparison to wild-type ctrl.: $P < 0.0001$. In (**E**), data of $n = 3$ excepting U2OS cells with $n = 6$ biological replicates. Significance values in comparison to ctrl.: HeLa cells (siRNA ctrl.): siRNA_1: $P = 0.0514$, siRNA2: $P = 0.4077$, siRNA ctrl.+ heparin: $P < 0{,}0001$; NIH 3T3 cells (wild-type ctrl.): NIH 3T3 cells + heparin: $P = 0.0016$, NIH 3T3 WASH KO: $P = 0.7838$; U2OS cells (wild-type ctrl.): U2OS cells + heparin: $P < 0{,}0001$, U2OS WASH KO: $P = 0.1849$. In (**F**), CD151 KO: $P < 0{,}0001$, EGFR KO: $P = 0.0156$. In (**G**), data of $n = 3$ biological replicates. Significance values in comparison to ctrl.: siRNA_1: $P < 0{,}0001$, siRNA_2: $P = 0.0009$, ctrl. + CytoD: $P < 0{,}0001$. In (**H**), data of $n = 3$ biological replicates. Significance values in comparison to ctrl.: SWIP – siRNA_1: $P = 0.0474$, siRNA_2: $P = 0.6608$; Strumpellin - siRNA_1: $P = 0.0207$, siRNA_2: $P = 0.3192$; CCDC53 – siRNA_1: $P = 0.0621$, siRNA_2: $P = 0.6655$; FAM21 - siRNA_1: $P = 0.0028$, siRNA_2: $P = 0.0148$.

 © The Author(s)

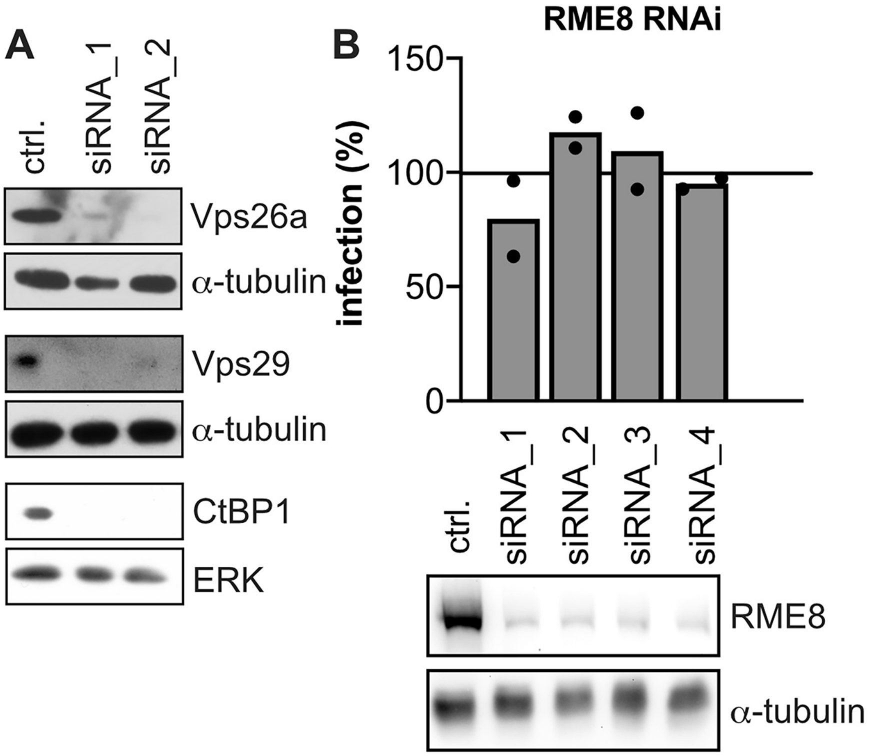

Figure EV4. RME-8 is dispensable for HPV16 infection.

(**A**) Depletion of Vps26a, Vps29, and CtBP1 by RNAi was confirmed by Western blotting. (**B**) RME8 was depleted from HeLa Kyoto cells by siRNA treatment and infected with HPV16-GFP. Infection was scored 48 h p.i. by automated microscopy and normalized to ctrl. Depicted is the mean of $n = 2$ biological replicates. Dots in the bar graph indicate the replicates. RME8 expression levels were analyzed by Western blotting.

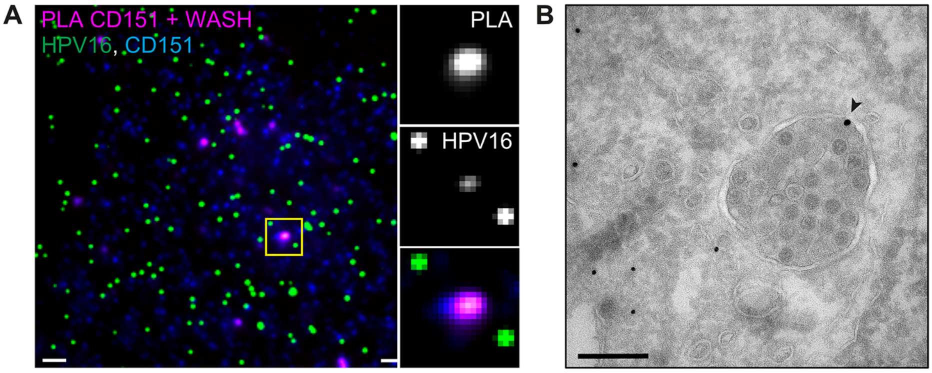

C

	binding	endocytosis	endosomes	ccv	nccv	nucleus	mitochondria	Σ
**GFP-WASH**	26	9	170	4	98	25	92	490
**ctrl.**	10	1	39	2	21	44	113	282
**fold enrichment**	2.6	9	4.4	2	4.7	0.5	0.8	-

Figure EV5. WASH is recruited to the plasma membrane.

(**A**) HPV16-AF488 was bound to ECM. HaCaT cells transfected with the HA-CD151 were seeded on top. Close proximity between WASH and HA-CD151 was detected using a PLA and plasma membrane localization was analyzed by TIRF-M. Scale bars are 2 μm and 0.5 μm. (**B**) Specific detection of EGFP-WASH on endosomes by immunogold labeling. Scale bar is 200 nm. (**C**) The GFP antibody specificity was assessed by quantification of the localization to indicated cellular compartments. Depicted is the absolute count of gold particles in 50 randomly selected profiles of EGFP-WASH expressing and untransfected (ctrl.) cells from $n = 2$ biological replicates (ccv clathrin-coated intracellular vesicle, nccv non clathrin-coated intracellular vesicle).

 © The Author(s)
